# Tranexamic acid reduces endometrial cancer effects through the production of angiostatin

**DOI:** 10.7150/jca.68169

**Published:** 2022-03-06

**Authors:** Keiichi Hiramoto, Yurika Yamate

**Affiliations:** Department of Pharmaceutical Sciences, Suzuka University of Medical Science, Suzuka, Mie, Japan

**Keywords:** Tranexamic acid, Endometrial cancer, Plasminogen, Macrophages, Angiostatin

## Abstract

Tranexamic acid (TA) has been reported to exhibit antitumor effects in various mouse models of cancer. However, the mechanism underlying its antitumor effects against endometrial cancer remains to be elucidated. This study was aimed at investigating the efficacy of TA against chronic inflammation-associated endometrial cancer induced by *N*-methyl-*N*-nitrosourea (MNU) and estradiol in a mouse model. After cancer induction, the mice were administered TA (12 mg/kg) three times weekly during the experimental period. The endometrial cancer development induced by MNU and estradiol was ameliorated by TA administration. Furthermore, TA treatment suppressed the levels of carbohydrate antigen 125, interleukin-6, and tumor necrosis factor-α in the plasma. The level of plasminogen, known as a TA target, increased in endometrial cancer and was further increased by TA treatment. On the other hand, plasmin levels increased in the model mice but decreased after TA treatment. Furthermore, the macrophage counts and the levels of matrix metalloproteinase (MMP)-12 and angiostatin in tumor cells in the uterus increased compared to the corresponding values in the control group and further increased upon TA treatment. The results of our study indicate that TA ameliorated the endometrial cancer induced by MNU and estradiol by regulating the macrophage/MMP-12/plasminogen/angiostatin signal transmission pathway.

## Introduction

The uterus is the organ in which the fetus grows during pregnancy. It is made up of muscle and covered by a mucous membrane called the endometrium. Uterine cancer is a general term for malignant tumors that develop in the uterus. The uterus is divided into a lower “cylindrical” portion and upper “cervical” portion of the “uterine body,” and the possible locations for development of tumors are grouped into “cervical cancer” and “endometrial cancer”, respectively. Endometrial cancer is caused by the long-term stimulation of the female hormone estrogen. It can be treated when detected early; however, no good preventive/therapeutic drugs have been found yet.

Tranexamic acid (TA: *trans*-4-aminomethylcyclohexanecarboxy acid) is an artificially synthesized amino acid that suppresses inflammation by inhibiting plasmin activity. Furthermore, TA acts on melanocytes, which produce melanin, and inhibits pigmentation and suppresses the development of spots and chloasma 1, 2. Furthermore, we found that TA administration improves skin wrinkles 3 and prevents osteoporosis 4. Thus, TA has diverse effects; nevertheless, its major therapeutic effect involves plasminogen. Angiostatin was discovered as a degradation product of plasminogen in 1994 5. Plasminogen is composed of linked kringle structures, and angiostatin is a fragment comprising one to four kringle structures of plasminogen 5. Angiostatin acts specifically on endothelial cells, suppressing endothelial cell proliferation, migration and angiogenesis 6-8. Angiostatin is not produced by a single enzyme but instead by various proteases from tumor cells 9-11. Thus, TA's effects may be related to angiostatin production, because it suppresses the synthesis of plasmin and fibrin from plasminogen. However, there are no reports available on the relationship between TA's anticancer effect and angiostatin. In this study, we investigated whether TA administration ameliorates endometrial cancer, and its relationship with angiostatin.

## Materials and Methods

### Animal experiments

**Figure 1:**
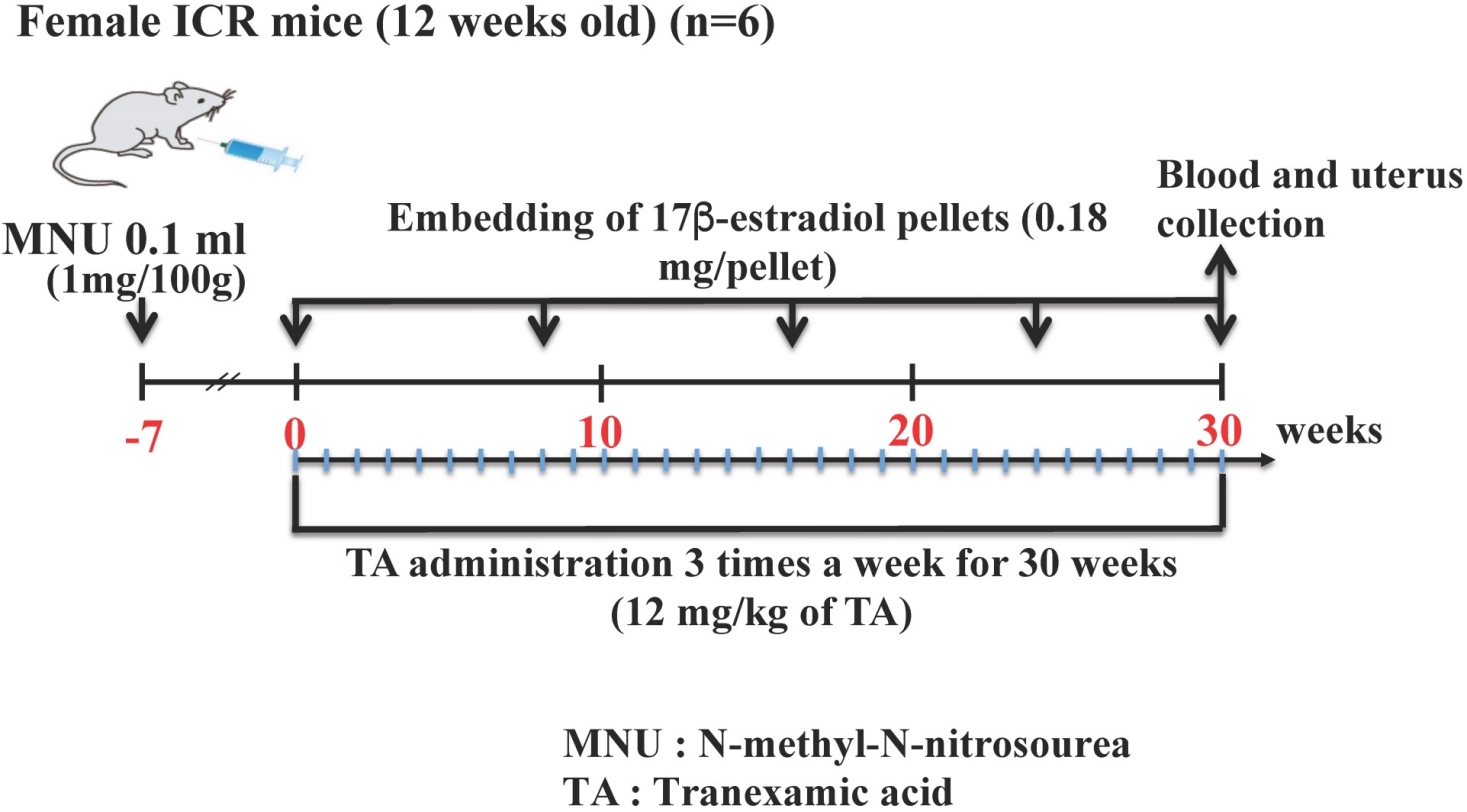
Schematic of the study procedure.

Twelve-week-old specific-pathogen-free (SPF) Institute of Cancer Research (ICR) mice were obtained from SLC, Hamamatsu, Shizuoka, Japan. All mice were individually housed in cages under a controlled temperature of 23°C ± 1°C and SPF conditions with a 12-h light/dark cycle (lights were turned on at 8:00 AM). Further, they were randomly divided into the following three groups with 10 mice in each group: control, endometrial cancer (treated with MNU and estradiol only), and TA-treated endometrial cancer groups. The animals were subjected to laparotomy under general anesthesia with pentobarbital sodium (Nembutal). *N*-Methyl-*N*-nitrosourea (MNU) solution (total volume: 0.1 ml) at a dose of 1 mg/100 g body weight was injected into the uterine tube. The control group was injected with saline into the uterine tube. One week after MNU exposure, the endometrial cancer and TA-treated endometrial cancer groups were administered 17β-estradiol. The use of 17β-estradiol treatment for endometrial cancer has been previously reported 12, 13. Briefly, MNU-treated mice were subcutaneously implanted with pellets that continuously released 17β-estradiol at doses of 0.18 mg over 60 days (Innovative Research of America Inc., Sarasota, FL, USA) in the dorsal back skin. The pellets were subcutaneously implanted three times during the 30-week study period. The time schedule for this experiment is shown in Figure 1. The study was strictly conducted according to the recommendations and guidelines for the care and use of laboratory animals at the Suzuka University of Medical Science (approval number: 34). All surgical procedures were performed under anesthesia with pentobarbital, and every effort was made to minimize animal suffering.

### TA treatment

The mice were orally administered approximately 12 mg/kg TA in 200 µL by sonde (Daiichi Sankyo Healthcare Co., Ltd., Tokyo, Japan) in distilled water three times per week for 30 weeks. The solvent-administered animals received distilled water 14.

### Preparation and staining of the uterus

Uterine samples were obtained on the final day of the examination. The uterine samples were fixed in phosphate-buffered paraformaldehyde (4%), embedded in frozen Tissue-Tek, an OCT compound, and cut into sections (thickness: 5 µm). The sections were stained with hematoxylin and eosin according to a protocol established for histopathological analysis 15. Additionally, the specimens were stained using an antibody for immunohistological analysis as described previously 16. The uterine specimens were incubated with a goat polyclonal anti-Ki67 (1:500; Santa Cruz Biotechnology, Santa Cruz, CA, USA) and a rat monoclonal anti-F4/80 (macrophage marker; 1:1000; Bio-Rad, Hercules, CA, USA) primary antibody. The specimens were subsequently incubated with fluorescein isothiocyanate-conjugated anti-rat or anti-goat secondary antibody (1:30; Dako Cytomation, Glostrup, Denmark). Macrophage count was evaluated immunohistochemically by fluorescence microscopy.

### Measurement of carbohydrate antigen 125, interleukin-6, tumor necrosis factor-α, plasminogen, plasmin, and tissue plasminogen activator (tPA) levels in the plasma

Blood samples were extracted from the hearts of the mice on the final day of the experiment. The plasma levels of carbohydrate antigen 125 (CA125), interleukin (IL)-6, tumor necrosis factor (TNF-α), plasminogen, plasmin, and tPA were determined using commercial ELISA kits (CA125: Bioassay Technology Laboratory, Shanghai, China; Il-6: Proteintech, Rosemont, IL, USA; TNF-α: R&D Systems, Minneapolis, MN, USA; plasminogen and plasmin: LSBio, Seattle, WA, USA; tPA: Abcam, Cambridge, UK) according to the respective manufacturers' instructions.

### Western blotting analysis of the uterus

The uterine samples were homogenized in a lysis buffer (Kurabo, Osaka, Japan). Then, the homogenates were centrifuged, and the resultant supernatants were collected. Western blotting was performed as described previously 3. Briefly, membranes were incubated at room temperature for 1 h with primary antibodies against plasminogen (1 : 1,000; GeneTex, Hsinchu City, Taiwan), plasmin (1: 1,000; GeneTex), tPA (1:1000; Abcam), matrix metalloproteinase (MMP)-12 (1: 1,000; Proteintech Rosemont, IL, USA), angiostatin (1: 1,000; Abcam), or β-actin as a loading control (1 : 5,000; Sigma-Aldrich, St. Louis, MO, USA). The membranes were then washed and incubated with horseradish peroxidase-conjugated secondary antibody (Novex, Frederick, MD, USA). Immune complexes were detected using ImmunoStar Zeta reagent (Wako, Osaka, Japan), and images were acquired using Multi Gauge software (Fujifilm, Greenwood, SC, USA).

### Statistical analysis

All data are presented as the mean ± standard deviation (SD) values. Microsoft Excel 2010 (Microsoft Corp., Redmond, WA, USA) was used to analyze the statistical significance of the data, along with one-way analysis of variance (ANOVA) followed by Tukey's post hoc test using SPSS version 20 (IBM, Armonk, NY, USA). Differences were considered significant at *p* < 0.05.

## Results

### Effect of TA on endometrial cancer induced by MNU and estradiol

**Figure 2:**
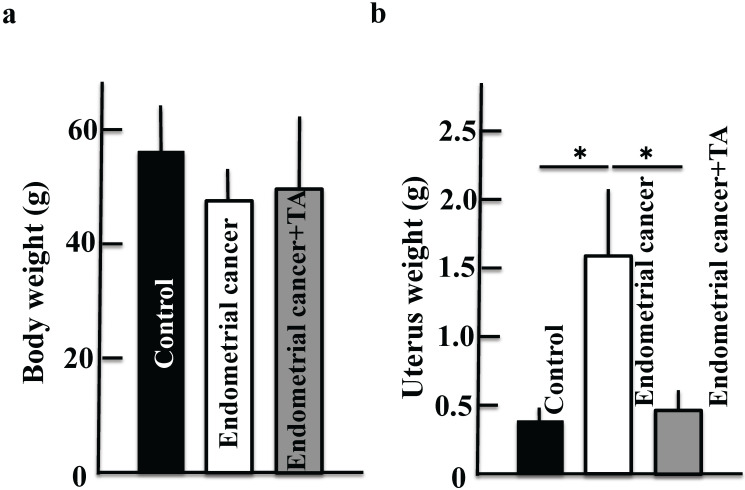

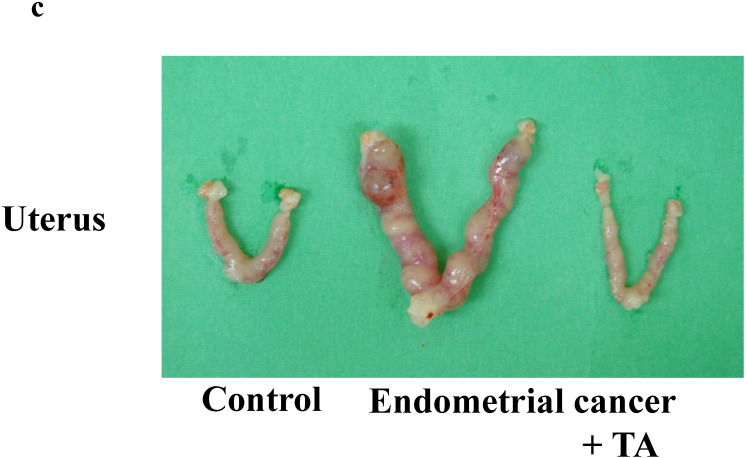

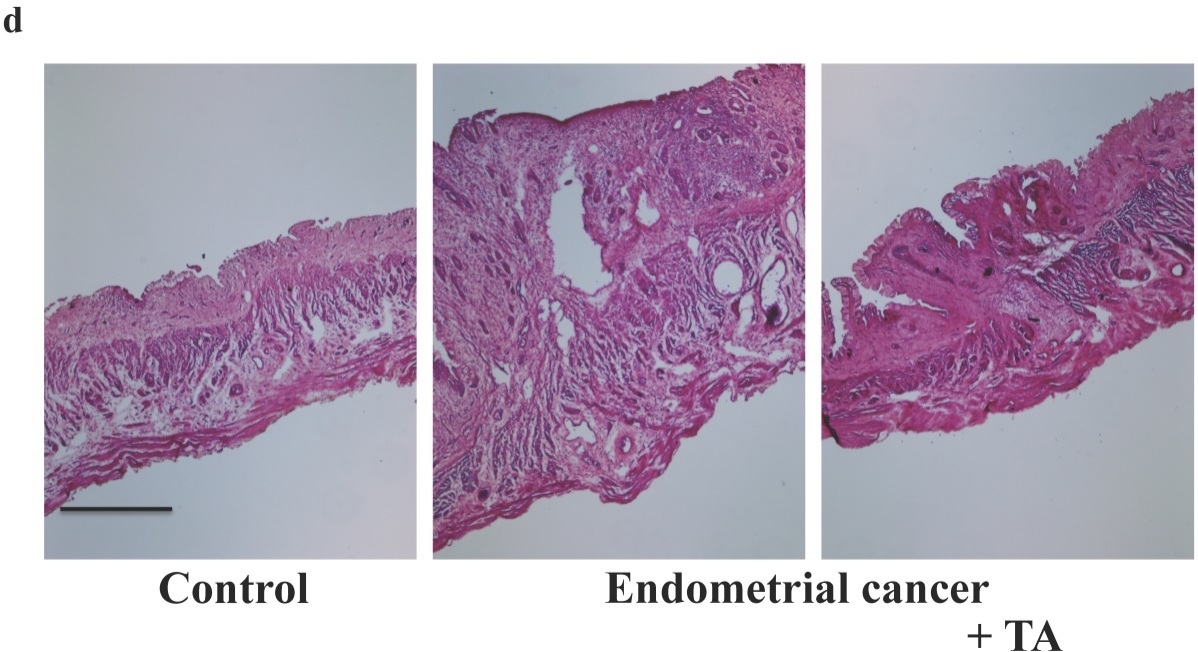

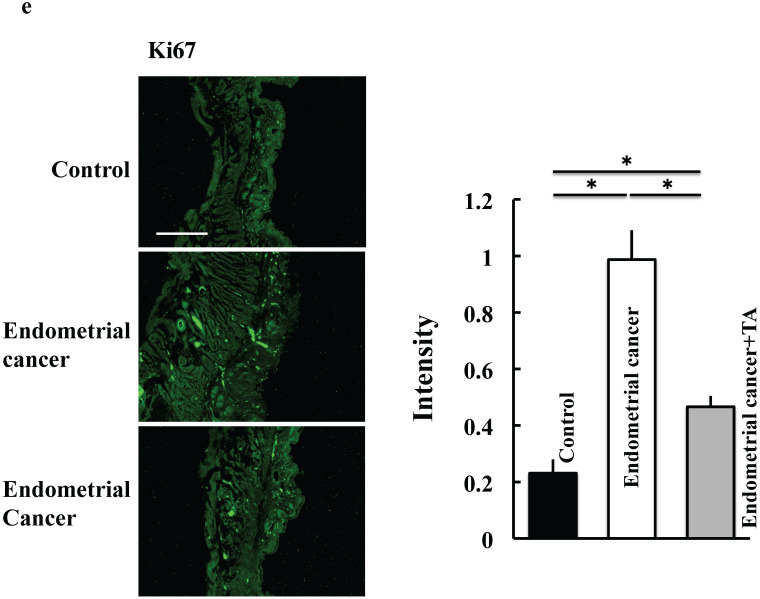
** Effects of TA treatment on endometrial cancer induced by MNU and estradiol. Thirty weeks after the start of the study, body weight (A),** uterus weight **(B)**, change of uterus **(C, D)**, and tumor cell marker (Ki67) **(E)** were measured. Values are expressed as the mean ± standard deviation (SD) derived from 10 animals. *p < 0.05. Scale bar = 100 µm. TA: tranexamic acid, MNU: *N*-methyl-*N*-nitrosourea.

TA (12 mg/kg) was administered orally three times per week for 30 weeks. On the final day of the experiment, the samples were observed for endometrial cancer microscopically. Compared to the endometrial cancer group, the TA-treated group showed improvements in the symptoms of endometrial cancer (Figure 2A-E).

### Effect of TA on CA125, IL-6, and TNF-α levels in the plasma

**Figure 3:**
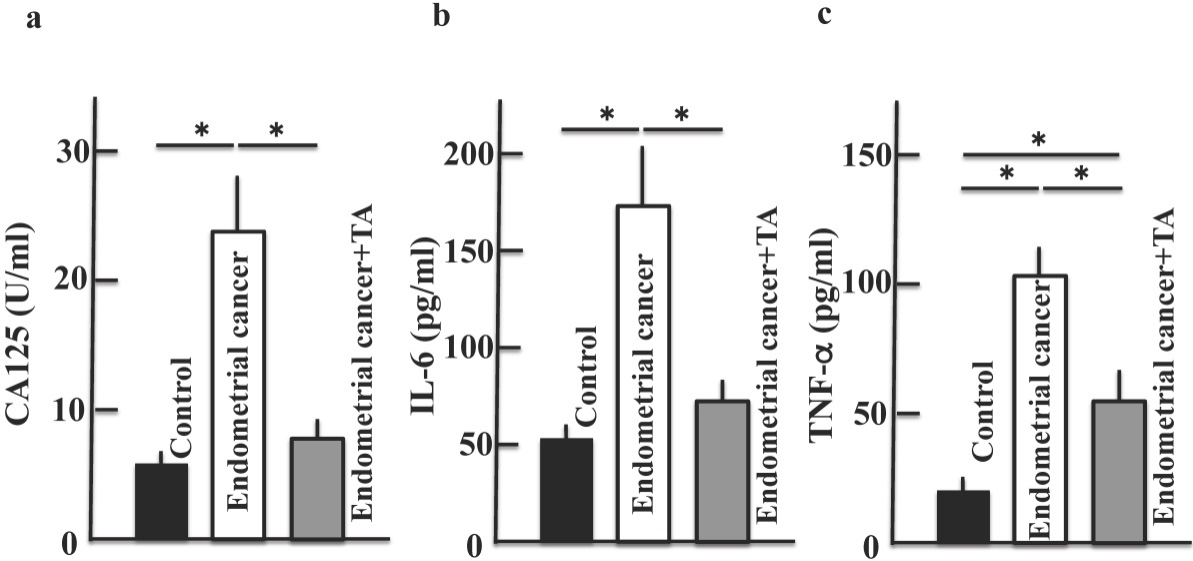
** Effects of TA treatment on the levels of CA125, IL-6, and TNF-α.** Thirty weeks after the start of the study, the levels of CA125 **(A)**, IL-6 **(B)**, and TNF-α **(C)** in the plasma of MNU- and estradiol-induced endometrial cancer model mice were measured. Values are expressed as the mean ± standard deviation (SD) derived from 10 animals. *p < 0.05. TA: tranexamic acid, MNU: *N*-methyl-*N*-nitrosourea, CA125: carbohydrate antigen 125, IL-6: interleukin-6, TNF-α: tumor necrosis factor-α.

We measured CA125, IL-6, and TNF-α levels to confirm the development of endometrial cancer. The plasma levels of CA125, IL-6, and TNF-α increased in the endometrial cancer group. However, treatment with TA decreased CA125, IL-6, and TNF-α levels (Figure 3A-C).

### Effects of TA on plasminogen, plasmin and tPA levels in the uterus and plasma

**Figure 4:**
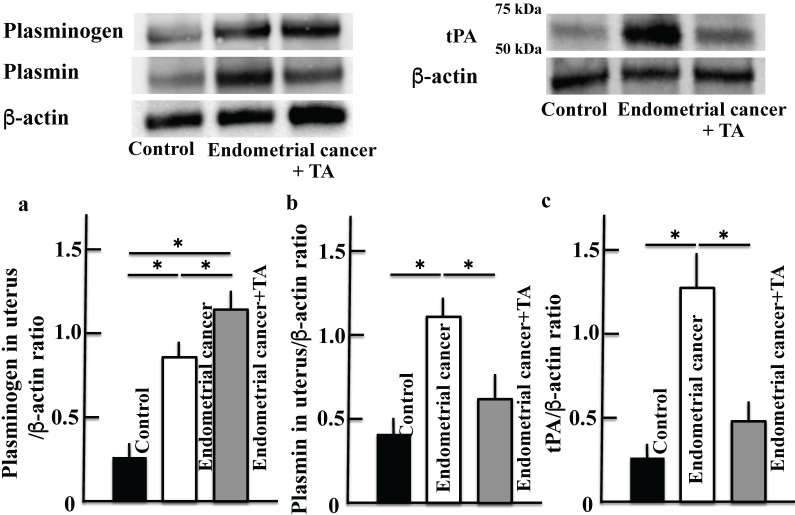

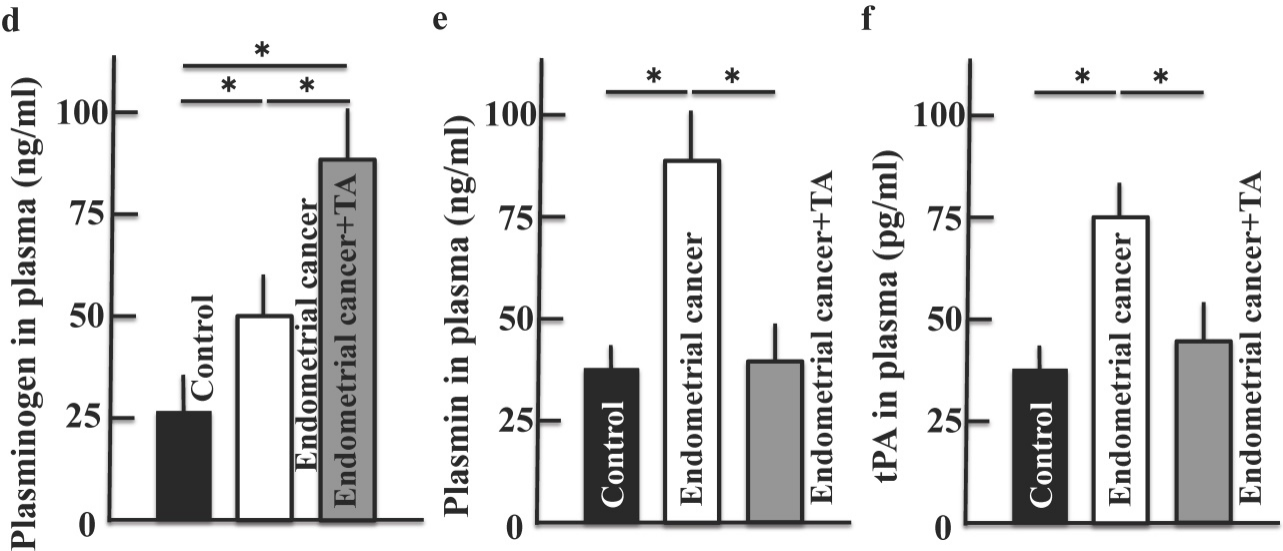
** Effects of TA treatment on plasminogen, plasmin and tPA levels.** Thirty weeks after the start of the study, by western blotting, the expression of plasminogen **(A)**, plasmin **(B)** and tPA **(C)** in uterus on MNU- and estradiol-induced endometrial cancer model mice were measured. By ELISA kit, the levels of plasminogen **(D)**, plasmin (E) and tPA (F) in plasma on MNU- and estradiol-induced endometrial cancer model mice were measured. Values are expressed as the mean ± standard deviation (SD) derived from 10 animals. *p < 0.05. TA: tranexamic acid, tPA: tissue plasminogen activator, MNU: *N*-methyl-*N*-nitrosourea.

We examined the levels of plasminogen (Figure 4A, D), plasmin (Figure 4B, E), and tPA (Figure 4C, F), which are the main targets of TA. Plasminogen, plasmin and tPA levels in the uterus and plasma were increased in the endometrial cancer group. The plasminogen level was further increased by TA treatment (compared to that in the MNU and estradiol groups). In contrast, the plasmin and tPA levels decreased to a level similar to that in the control group. In addition, the results of more three samples of plasminogen (a) and plasmin (b) are shown in the supplementary Figure 1.

### Effect of TA on the macrophage count in the uterus

**Figure 5:**
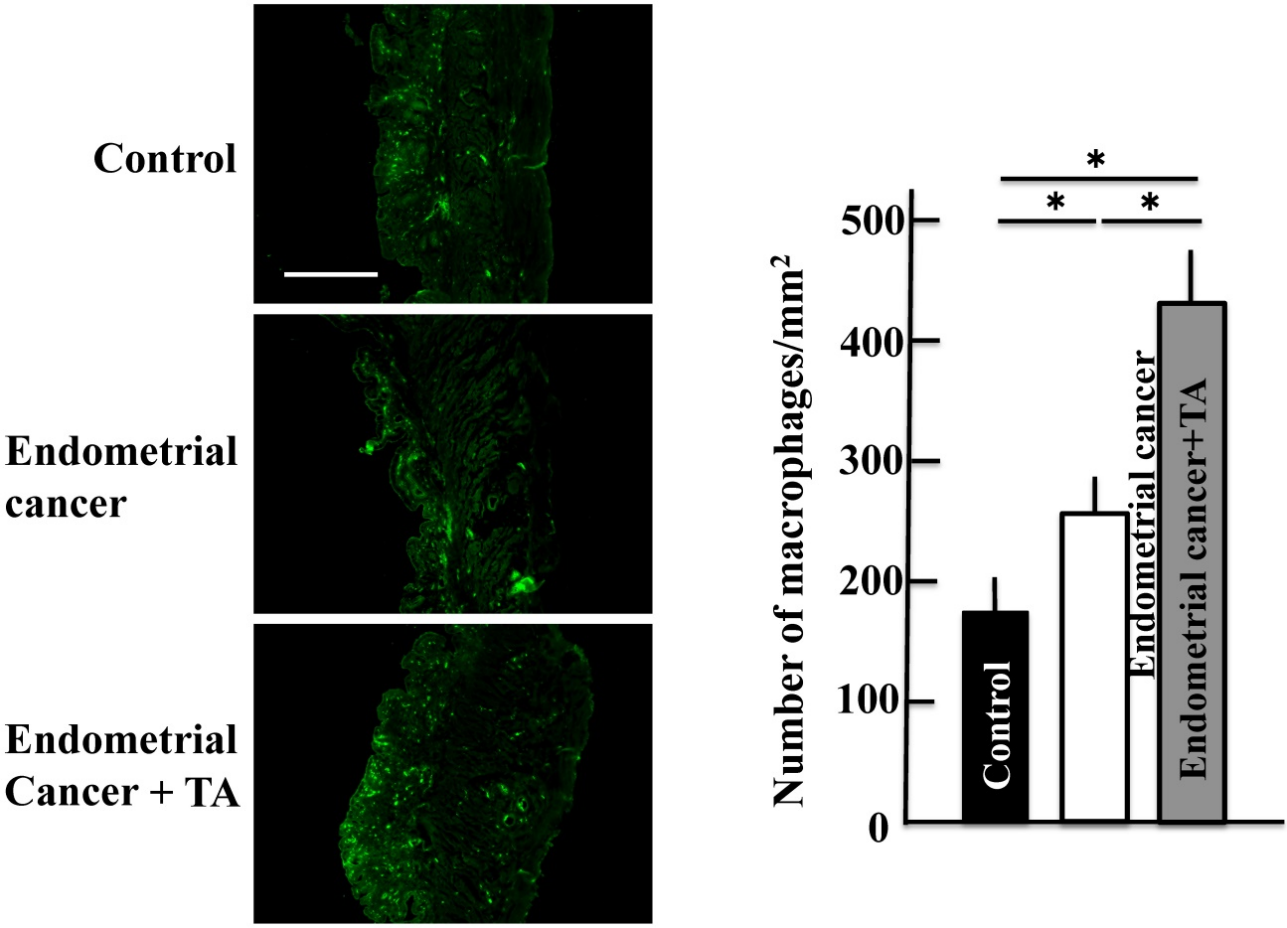
** Effects of TA treatment on macrophage count. Thirty weeks after the start of the study,** the macrophage count in the uterus of MNU- and estradiol-induced endometrial cancer model mice was determined. Values are expressed as the mean ± standard deviation (SD) derived from 10 animals. *p < 0.05. Scale bar = 100 µm. TA: tranexamic acid, MNU: *N*-methyl-*N*-nitrosourea.

The macrophage count increased in the group treated with only MNU and estradiol. The macrophage count further increased upon TA treatment compared to that in the MNU and estradiol groups (Figure 5).

### Effect of TA on MMP-12 and angiostatin levels in the uterus

**Figure 6:**
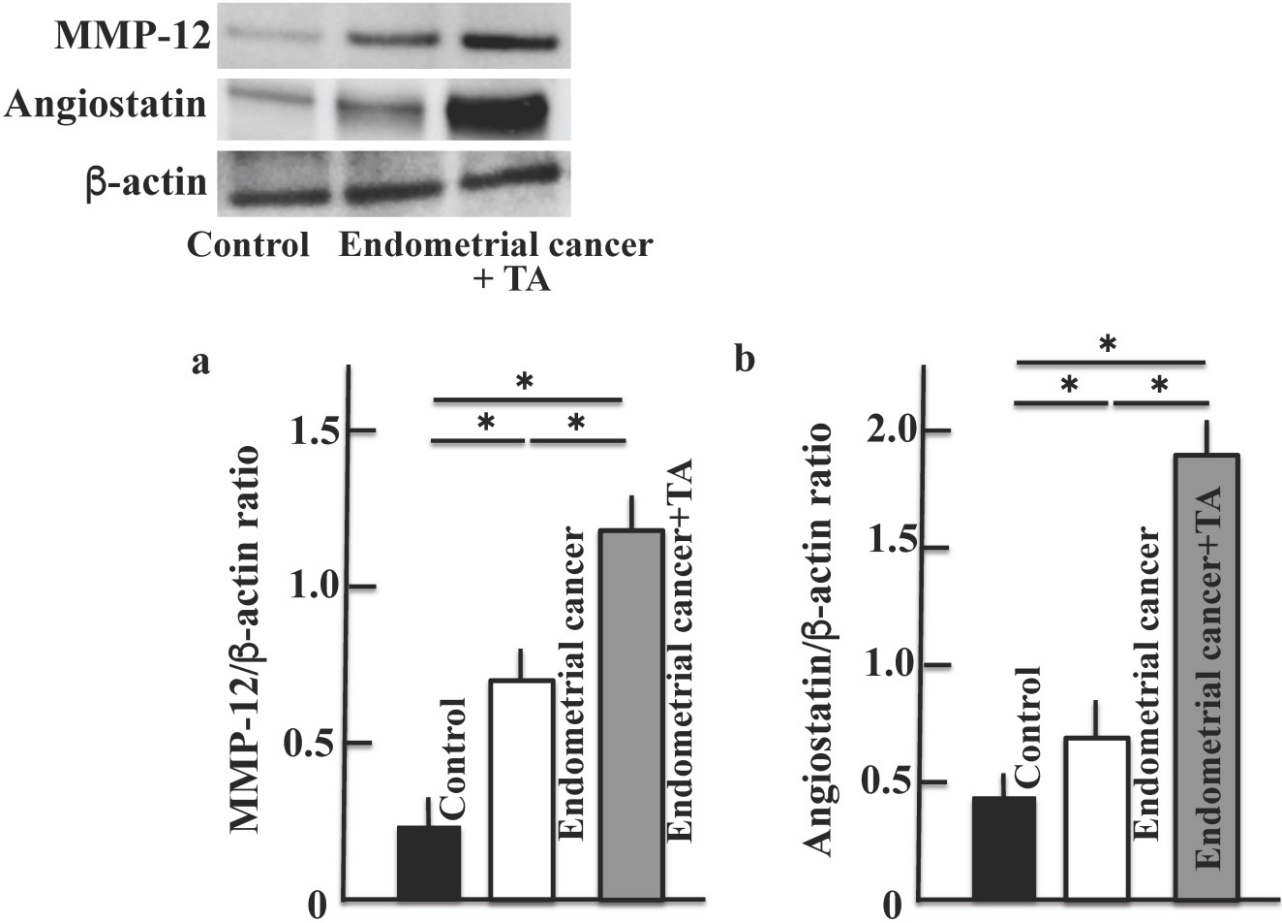
**Effects of TA treatment on MMP-12 and angiostatin levels.** Thirty weeks after the start of the study, MMP-12 **(A)** and angiostatin **(B)** levels in the uterus of MNU- and estradiol-induced endometrial cancer model mice were determined. Values are expressed as the mean ± standard deviation (SD) derived from 10 animals. *p < 0.05. TA: tranexamic acid, MNU: *N*-methyl-*N*-nitrosourea, MMP: matrix metalloproteinase.

Next, we measured the levels of MMP-12 secreted by macrophages (Figure 6A) and angiostatin produced from plasminogen (Figure 6B). The MMP-12 and angiostatin levels increased in the group treated with only MNU and estradiol. The levels of both MMP-12 and angiostatin were further increased upon TA treatment compared to the corresponding levels in the MNU and estradiol groups. In addition, the results of more three samples of MMP-12 (c) and angiostatin (d) are shown in the supplementary Figure 1. Then, the difference in molecular weight was shown by inserting a molecular weight marker in the Western blot figures of plasminogen (a), plasmin (b) and angiostatin (c) (Supplementary Figure 2).

## Discussion

In this study, we found that treatment with TA reduced the effects of endometrial cancer, which were induced by MNU and estradiol. Furthermore, it resulted in an increase in plasminogen and a decrease in plasmin levels, which were increased by the tumor. In addition, TA treatment resulted in an increase in the macrophage count and MMP-12 level, thereby inducing an increase in the level of angiostatin produced by plasminogen.

Tumor cells increase the production of plasminogen, activate the serine protease plasminogen activator, and induce the conversion of plasminogen to plasmin. Plasminogen is involved in the infiltration and metastasis of malignant tumors via the activation of pro-collagenase and abduction of the extracellular matrix 17. Tumor cells also accumulate macrophages. It has been reported that in this reaction mechanism, tumor cells activate the mechanistic target of rapamycin complex 1 (mTORC1)-forkhead box K1 (FOXK1)-C-C motif chemokine 2 (CCL2) pathway and cause the accumulation of tumor-associated macrophages 18. Macrophages infiltrating the tumor express MMP-12, which degrades plasminogen to produce angiostatin 11. Angiostatin is known to suppress the proliferation 7 and migration 8 of endothelial cells and inhibit angiogenesis. In this study, administration of TA suppressed plasminogen activation (Figure 4), and plasminogen produced by tumor cells was not activated and accumulated. At the same time, tumor cells express a large amount of MMP-12 by accumulating macrophages. This leads to the degradation of plasminogen by MMP-12 to produce a large amount of angiostatin, which has an antitumor effect (Figure 6).

In this study, we investigated the therapeutic effect of TA in a mouse model of endometrial cancer. The expression of vascular endothelial growth factor was found to be the reason for the effectiveness of TA in endometrial cancer. Placental growth factor (PlGF), a subtype of vascular endothelial growth factor (VEGF), is expressed in the uterus and maintains pregnancy 19, 20. The development of endometrial cancer increases PlGF expression in the endometrium. However, the administration of TA signify reduced PlGF expression, leading us to conclude that the angiostatin level increased by TA treatment suppressed PlGF expression in the endometrium. However, the mechanism by which angiostatin induces PlGF suppression is unknown and requires further investigation.

## Conclusion

**Figure 7:**
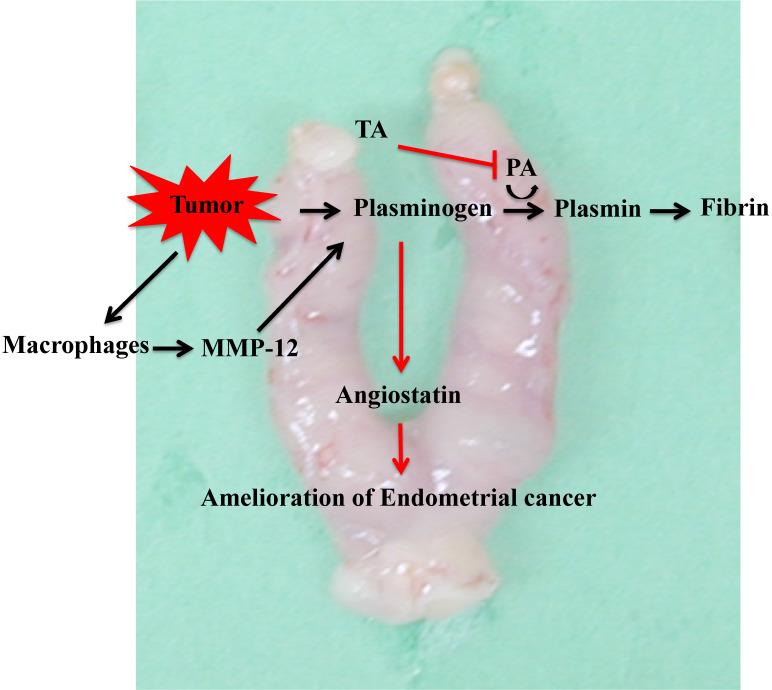
Mechanism underlying the effect of TA in the MNU- and estradiol-induced endometrial cancer model mice. TA: tranexamic acid, MNU: *N*-methyl-*N*-nitrosourea

In this study, we found that TA suppressed the onset of endometrial cancer. The mechanism underlying this action by TA involved the inhibition of plasminogen activator, thus resulting in an increase in plasminogen. At the same time, MMP-12 produced by macrophages that infiltrate tumor cells degrades plasminogen to produce angiostatin. The anti-angiogenic effect of angiostatin ameliorated endometrial cancer effects (Figure 7). The results of this study indicate that TA is effective not only against endometrial cancer but also against other fixed cancers, and we are investigating its efficacy against other cancers. Nevertheless, we used a mouse model of MNU- and estradiol-induced endometrial cancer in this study. Therefore, it is uncertain whether this effect could be observed in humans, and in the future, it will be necessary to conduct clinical trials in humans.

## Supplementary Material

Supplementary figures.
