# Supplementary material for: Tranexamic acid reduces endometrial cancer effects through the production of angiostatin
Source: J Cancer. 2022 Mar 6;13(5):1603–10. doi: 10.7150/jca.68169 (PMC8965126; doi:10.7150/jca.68169)
Supplement: Supplementary file 1 — Supplementary figures. [file jcav13p1603s1.pdf]

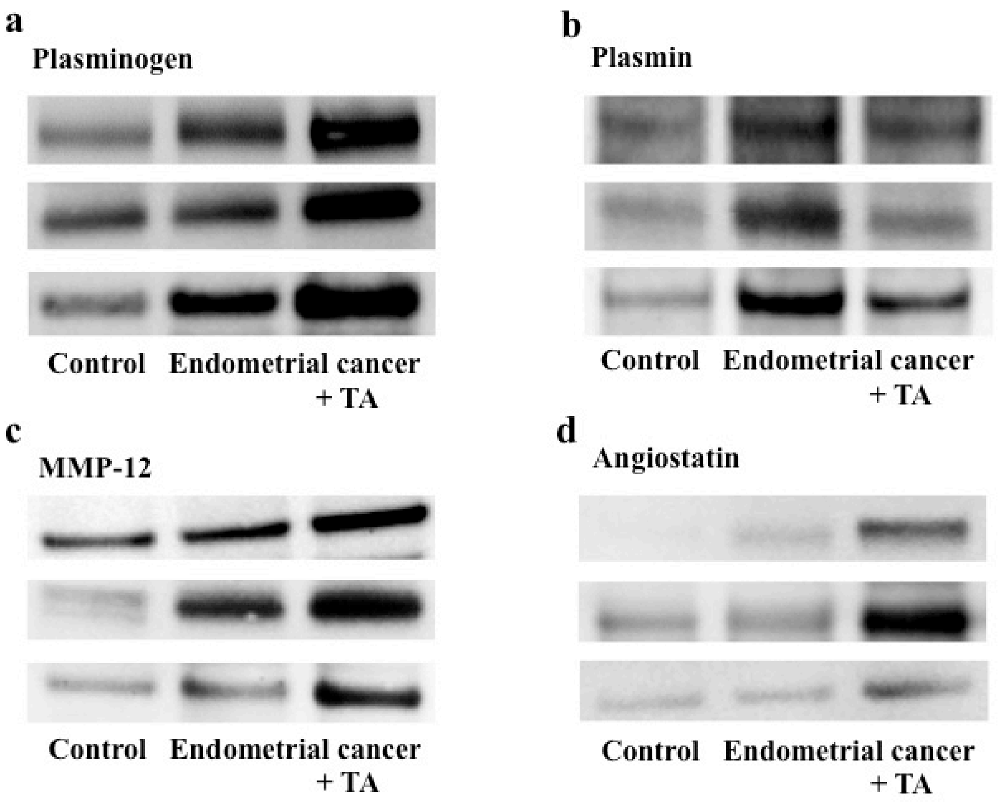

Supplementary Fig. 1 The results of more three samples of plasminogen (A), plasmin (B), MMP-12 (C) and angiostatin (D). TA: tranexamic acid, MMP: matrix metalloproteinase.

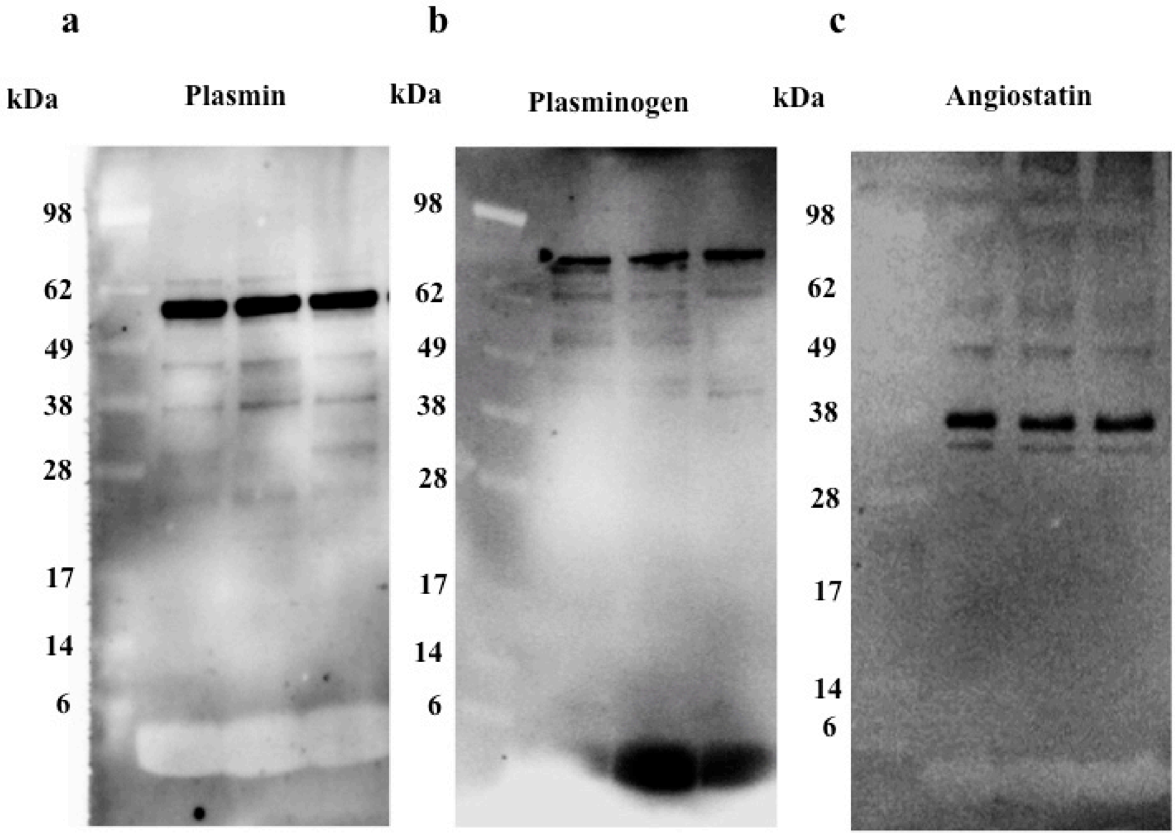

Supplementary Fig. 2 Western blot diagram of plasminogen (A), plasmin (B) and angiostatin (C) with moleculer weight markers. All lanes were flowed tumor cell extraction samples.
